# Brain-Computer Interface for Clinical Purposes: Cognitive Assessment and Rehabilitation

**DOI:** 10.1155/2017/1695290

**Published:** 2017-08-23

**Authors:** Laura Carelli, Federica Solca, Andrea Faini, Paolo Meriggi, Davide Sangalli, Pietro Cipresso, Giuseppe Riva, Nicola Ticozzi, Andrea Ciammola, Vincenzo Silani, Barbara Poletti

**Affiliations:** ^1^Department of Neurology and Laboratory of Neuroscience, IRCCS Istituto Auxologico Italiano, P.le Brescia 20, 20149 Milan, Italy; ^2^Department of Cardiovascular, Neural and Metabolic Sciences, IRCCS Istituto Auxologico Italiano, P.le Brescia 20, 20149 Milan, Italy; ^3^ICT & Biomedical Technology Integration Unit, Centre for Innovation and Technology Transfer (CITT), Fondazione Don Carlo Gnocchi Onlus, Via Capecelatro 66, 20148 Milan, Italy; ^4^Applied Technology for Neuro-Psychology Lab, IRCCS Istituto Auxologico Italiano, P.le Brescia 20, 20149 Milan, Italy; ^5^Department of Psychology, Catholic University of Milan, Largo A. Gemelli 1, 20123 Milan, Italy; ^6^Department of Pathophysiology and Transplantation, “Dino Ferrari” Center, Università degli Studi di Milano, Via Francesco Sforza 35, 20122 Milan, Italy

## Abstract

Alongside the best-known applications of brain-computer interface (BCI) technology for restoring communication abilities and controlling external devices, we present the state of the art of BCI use for cognitive assessment and training purposes. We first describe some preliminary attempts to develop verbal-motor free BCI-based tests for evaluating specific or multiple cognitive domains in patients with Amyotrophic Lateral Sclerosis, disorders of consciousness, and other neurological diseases. Then we present the more heterogeneous and advanced field of BCI-based cognitive training, which has its roots in the context of neurofeedback therapy and addresses patients with neurological developmental disorders (autism spectrum disorder and attention-deficit/hyperactivity disorder), stroke patients, and elderly subjects. We discuss some advantages of BCI for both assessment and training purposes, the former concerning the possibility of longitudinally and reliably evaluating cognitive functions in patients with severe motor disabilities, the latter regarding the possibility of enhancing patients' motivation and engagement for improving neural plasticity. Finally, we discuss some present and future challenges in the BCI use for the described purposes.

## 1. Introduction

BCIs have been studied with the primary motivation of providing assistive technologies for people with severe motor disabilities, particularly locked-in syndrome (LIS) caused by neurodegenerative disease such as Amyotrophic Lateral Sclerosis (ALS) or by stroke [1]. Such approach involves the use of suitable cortical signals as input to control external devices or for Augmentative and Alternative Communication purposes in patients suffering from central nervous system injury. BCI has been studied for more than 25 years and has been extensively validated, even if with still heterogeneous results according to both the method employed and the populations involved [2, 3]. A review of BCI studies is not within the objective of the present work [4].

A newly emerging field of research concerns the use of BCIs to enhance motor and cognitive recovery within neurorehabilitation settings. In fact, most of common rehabilitation tools require a minimal level of motor control to perform the therapeutic tasks; therefore, patients with severe motor deficits are not allowed to accomplish traditional rehabilitation training. Some recent reviews have presented and discussed main advances in the use of BCIs for rehabilitation purposes [5–7]. A further work has discussed the current status of BCI as a rehabilitation strategy in stroke patients [8]. In addition to the use of BCI to restore motor function or provide feedback to patients (i.e., during motor imagery), the authors underlie further advantages of brain activation monitoring during rehabilitation, in particular, the possibility of monitoring the global level of attention concerning the task and the level of interhemispheric balance.

Within the neurorehabilitation setting, the assessment and training of cognitive impairments represent a more innovative and less explored area. The evaluation of cognitive abilities in patients at advanced stages of paralysis represents a challenge, since standard assessment tools for both verbal and nonverbal cognitive abilities typically involve a motor response. In ALS, evidence suggests the need for some task modifications in order to make the standard neuropsychological assessment suitable for patients with verbal and motor impairment [9]. Also the* Edinburgh Cognitive and Behavioural ALS Screen* (ECAS), recently designed by Abrahams and colleagues [10] for ALS patients, cannot be performed in moderate-severe stages of the disease. Besides, even tests relying on some form of rudimentary motor function such as blinking, nodding, or pointing [11] are not administrable to totally locked-in patients where even the presence of minimal motor functions could be prevented.

Recently, some attempts have been made in order to obtain verbal-motor free indicators of executive functions changes in ALS. In particular, event-related potentials (ERP) have been employed to assess cognitive dysfunctions with minimal motor demands [12–14]. Such approach, even if valuable, provides quantitative and qualitative data not comparable with scores obtained from standard cognitive testing, therefore not allowing a reliable and longitudinal evaluation of neuropsychological functions.

The evaluation of cognitive capacities in patients with severe motor disabilities has also relevant implication for BCI systems usability aspects. Among the physiological and psychological factors that influence or affect BCI use, several studies have showed an effect of both general mental load and more specific cognitive functions on BCI performance. For example, the P300 ERP signals, employed in the frequently adopted P300 BCI systems, depend on attention and working memory processes; in such approach, reduced level of attention or higher levels of working memory load are associated with lower amplitudes and prolonged latencies [15]. Recent studies confirmed the role of working memory, together with general intelligence [16] and attention [17], on P300 BCI performance. Some approaches have attempted to manage such aspects by employing different interfaces [18] or stimulation modes [19], in order to reduce mental load. In addition to the need for technical adaptations, the described findings suggest the potential benefit of working memory training to improve BCI usability and performance.

The use of BCIs for cognitive training is another emerging field of study within neurorehabilitation settings and could improve both patients' clinical conditions and BCIs' usability. In particular, the possibility of enhancing neural plasticity by providing real-time feedbacks in an engaging setting could improve the treatment efficacy and transferability to real-life contexts.

We present the current state of art about BCI applications addressing cognitive aspects, with regard to approaches targeting both assessment and rehabilitation of cognitive functions. As below described, such approaches involve patients with severe motor deficits, in order to overcome verbal-motor limitations, together with other clinical populations without physical disability, according to the advantages provided by the use of BCI with respect to traditional cognitive training methods.

## 2. Material and Methods

Between January and February 2017 we performed a search on the PubMed, Web of Science, and Scopus databases. We searched the terms “BCI” or “brain-computer interface” or “brain machine interface” in combination with the following terms: “neurofeedback,” “cognitive,” “rehabilitation,” “training,” “assessment,” and “neuropsychological.” Other definitions of BCI (i.e., Mind-Machine Interface (MMI)) were included; however no relevant results were obtained, according to the topic of the present review. We searched the reference list of retrieved papers to identify additional relevant articles. Only studies in English were considered for the present systematic review. Other reviews of literature dealing with the topic of our work have been considered within this work. A total number of 1701 items have been found with PubMed, 2950 items with Web of Science, and 3977 items with Scopus.

Studies where NF was described without referring to a BCI system were excluded. Moreover, studies were cognitive tasks/abilities were included in the BCI protocols with aims other than assessment or rehabilitation of cognitive abilities (i.e., monitoring of cognitive state during motor rehabilitation, means to perform motor tasks; study of brain functions not aimed at clinical purposes) were not considered.

The systematic search resulted in 9 records for cognitive assessment and 15 records for cognitive training, consisting of experimental studies that were included in Tables 1 and 2. Studies presenting design or development of BCI-based protocols without reporting experimental data on healthy controls or clinical populations were considered within the manuscript, but not reported in the Tables. Other results concerning NF and BCI studies or reviews have been considered and reported within introduction and discussion for enhancing and supporting considerations about the described results. Actually, to the best of our knowledge, no other review concerning BCI use for cognitive assessment or rehabilitation is available.

## 3. Results and Discussion

### 3.1. Cognitive Assessment through BCI-Based Systems

The application of BCI systems in order to develop new neuropsychological assessment tools mainly employed EEG-based BCIs (see Table 1). These types of BCI are included in noninvasive BCIs; that is, they do not require surgical implantation to acquire signals; surface EEG is the most widely used noninvasive technique for BCI studies in neurological patients [20]. The main EEG-based paradigms detected and used are sensorimotor rhythms (SMRs), slow cortical potentials (SCPs), event-related potentials (ERPs), and visually evoked potentials (VEPs).

Iversen et al. [21, 22] aimed at assessing some cognitive functions in completely paralyzed ALS patients by developing a SCPs EEG-BCI. In a first study [21], training was applied to two severely paralyzed ALS patients, during which they could learn to control certain components of their EEG in order to direct the movement of a visual symbol on a monitor. Next, a series of two-choice cognitive task was administered, such as odd/even number and larger/smaller numbers discrimination. Performance was also assessed using a matching-to-sample paradigm, which was used to examine the ability to discriminate numbers, letters, colors, and to perform simple calculations. In a successive study, Iversen et al. [22] employed the same SCP-EEG control in order to administrate a conditional-associative learning task to a late-stage ALS patient, testing the ability to learn arbitrary associations among visual stimuli. In both studies, a good level of accuracy was observed in detecting patients' performances, according to a within subjects experimental design. Patients were also able to understand the verbal instructions and to respond accordingly in the successive tasks. However, such method requires an extensive pretraining in order to learn to control EEG, which can take some weeks; moreover, it cannot be used for tasks based on recall or where a choice must be made among more than two stimuli.

Perego and coll. [23] applied a steady state visually evoked potentials (SSVEP) based BCI system to develop a psychometric assessment based on a widely used clinical test (Raven Colored Progressive Matrices (RCPM)). The protocol has been validated on 19 healthy subjects and compared to a paper-based administration: results showed congruent performances obtained with the two methods. A successive study by the authors [24] tested the SSVEP BCI cognitive protocol on a sample of patients with physical disabilities due to different neurological disease and confirmed its reliability in a clinical population; however, 11 out of 26 participants were excluded from the protocol according to involuntary movements and poor cooperation or because they did not elicit SSVEP response. Westergren et al. [25] applied a SSVEP-based BCI to develop four cognitive tests based on the Wechsler Adult Intelligence Scale (WAIS) matrix tests; they administered the short battery to a group of 11 healthy subjects, obtaining findings that supported the accuracy and usability of the developed system. Even if promising, this protocol should be validated on a clinical population.

Overall, the described approaches present some limitations, such as important rearrangement of the original cognitive tests, possibly producing biased results and extensive pretraining; furthermore, the adaptation of single cognitive tests does not match the clinical need for a comprehensive neuropsychological evaluation.

Differently from other BCIs approaches, P300-based ones do not require learning of self-regulation of the brain response and feedback. A possible reduction in training time represents an important chance in order to extend the use of Augmented and Alternative Communication (AAC) to cognitive assessment purposes [26]. On the other side, the use of P300 requires, as a precondition, an intact visual system, at least for the visual modality, which has been proved to be more reliable than the auditory one and preserved ability to pay attention; this may represent a problem in some patients. Recently, we presented a new verbal-motor free neuropsychological battery, by adapting some traditional neuropsychological tests (i.e., Token Test, Modified Card Sorting Test (MCST), Raven Colored Progressive Matrices (RCPM), and d2 Test) to the P300-BCI administration, according to a reasonable adherence to the original validated tests [27]. Usability components, relationship to clinical and psychological variables, and convergent validity of the developed battery in a sample of ALS patients and healthy controls were investigated. In ALS patients, the proposed P300-BCI-based assessment showed a high rate of calibration accuracy, together with satisfactory levels of usability and sensitivity, independently from clinical aspects, such as disease progression (ALFRS-R) and disease onset, or psychological factors such as anxiety and depression. Even if the described protocol satisfies the need for a comprehensive evaluation of cognitive abilities, some issues arise from performing several tests with BCI; in particular, prolonged time for administration and cognitive effort could involve fatigue effects and reduce the reliability of the assessment. This study was included within an extended project, evaluating P300-BCI use for neuropsychological assessment with a particular attention to usability, pleasantness, fatigue, and emotional aspects [28, 29]. Within such project, preliminary attempts to adapt another widely used traditional neuropsychological test, that is, Verbal Fluency, have been performed as a proof of concept which needs further investigations.

Recently, an hybrid brain-computer interface combining P300 and SSVEP has been used to detect number processing and mental calculation in patients with disorder of consciousness (DOC) [30]. Results were obtained on eleven patients: five of them achieved accuracy rates that were significantly higher than the chance level and demonstrated preserved ability to follow commands, in addition to number processing and calculation abilities. However, patients were easily fatigued, thus leading to insufficient training data, and their level of object-selective attention was much lower than for healthy subjects. Moreover, gaze-dependent BCIs can provide unreliable data in DOC patients, since they often lose their ability to fixate their gaze; therefore, visual abilities should be accurately evaluated when employing gaze-dependent BCIs that should eventually be replaced by gaze-independent systems.

Then, the field of research about the development of cognitive tasks based on BCI for patients with motor disabilities is still at dawn and represents a promising area to be developed.

### 3.2. Cognitive Training in Neurological Patients and Healthy Subjects by Means of BCI

BCI has been used to enhance attention and other cognitive abilities (see Table 2), based on the principle of neurofeedback (NF) therapy (T). In particular, a largely employed NFT is that based on surface EEG, as it is relatively cheap, usable, and portable. EEG-NFT involves that neural signals can be measured and used to improve neural functions: patients observe a suitable graphical representation of their actual brain activity, usually processed through a computer, and learn to self-regulate this activity in order to bring it to a desired state. This approach has been used for treating several conditions, including both neurological and psychological disorders, such as attention-deficit hyperactivity disorder (ADHD), anxiety, epilepsy, and addictive disorders [31]. Moreover, NF has also been applied for cognitive enhancement [32–34]. Typically, tasks involved in NFT are repetitive and standardized and respond to the need to indicate to participants when they have reached the required brainwave pattern. Some of results obtained employing such approach with ADHD patients are controversial; for example, a recent systematic review and an experimental study [35] concluded that literature fails to support any benefit of NF on neurocognitive functioning in ADHD, possibly due to study limitations. An extensive review on NF approaches is beyond the scope of this article.

Recently, EEG-BCI systems have been employed in order to improve cognitive functions in patients with ADHD. Munoz and colleagues [36] designed and presented a BCI-based videogame for training sustained attention in ADHD patients, to be implemented by means of low-cost BCI systems. However, such system has not been validated in a clinical population. Lim and colleagues [37, 38] developed a series of training games, where users' attentional levels measured by EEG signals can be used to perform exercise. Such approach proved to be useful to enhance attention abilities in children with ADHD, by improving parent-rated inattentive scores on the ADHD Rating Scale.

Lee and colleagues then modified their training program in a successive study, introducing a new game with a memory training component addressing elderly population [39]. The BCI training was showed to improve both attention and visuospatial and memory components; moreover, usability and acceptability rates were satisfying for the target population. The same authors then replicated the study on a sample of healthy, predominantly Chinese-speaking elderly, in order to determine the generalizability of the developed system and training task to a different linguistic population [40]. They confirmed the BCI training potential in improving cognition in both English- and Chinese-speaking elderly, showing its usability and acceptability in the latter population.

Another application of BCI for cognitive enhancement in the elderly has been developed by Gomez-Pilar and colleagues [41, 42]. The authors developed a motor-imagery-based BCI system to perform NF in healthy elderly, which was realized by means of five different tasks of increasing difficulty levels. In such tasks, subjects were trained in learning and practice motor imagery and performing logical and memory exercises. Feedback consisted of an item moving on the screen, controlled by motor imagery tasks. Results from cognitive tests and EEG changes showed an improvement after five sessions. In particular, cognitive changes concerned visuospatial, language, memory, and conceptual domains.

Such studies are of particular interest, because only few application of NFT previously addressed cognitive enhancement in the elderly [31, 43, 44].

Pineda et al. [45] also hypothesized that BCI-based NF using specific EEG frequency bands should induce neuroplastic changes of the mirror neuron system in autism spectrum disorder (ASD). According to these suggestions, Friedrich and colleagues [46] developed a BCI game application for combined NF and biofeedback treatment of children with ASD. The proposed system requires children to modulate their brain activity and peripheral physiological activation in social games, with feedback consisting in emotional imitation behavior within social interactions. Such approach entails the value of maintaining player interest and realizing ecological situations in order to maximize learning and transferability to real-life contexts.

Kim & Lee [47] employed a BCI-based functional electrical stimulation (FES) training on children affected by spastic cerebral palsy, with EEG patterns during concentration used to trigger FES: FES was applied as patients concentrated on finger extension, wrist extension, wrist abduction, and wrist circumduction while holding a wrist bar. SMRs and middle beta waves (M-beta) were recorded prior and after the training as outcome measures. Results showed an increase of such indexes that are associated with logical thinking, problem solving, and attentiveness to external stimuli, suggesting an effect of the performed training also on nonmotor functions. Salisbury and colleagues [48] presented a single-case feasibility study on a patient with spinal cord injury (SCI), where EEG-BCI was employed for reducing pain and improving nonmotor functions such as mood and cognition, as part of inpatient rehabilitation treatment. Even if no data are presented about the described therapeutic goals, the study supported the feasibility and tolerability of this approach in the patient. A successive study on an extended sample of SCI patients [49] did not show any effect of the BCI training on measures related to cognition, psychological disposition, and pain.

Another application of BCI to recovery cognitive functions regards aphasia rehabilitation in stroke patients [50, 51]. Kleih and colleagues [50] supported the feasibility of a P300-BCI speller communication system with aphasic patients, after implementation of individualized adaptations and some training. According to the authors, further application of the developed approach could involve improvement of neural plasticity by activating language circuits, promoting aphasia recovery. Musso and colleagues [51] preliminary investigated the presence of neuronal markers of auditory attention and acoustic processing as prerequisite for auditory BCI application in a stroke patient and concluded that BCI training could be feasible for him. Such promising findings should be supported by further investigations on the target sample, but could be preliminary to BCI applications for rehabilitation of speech production deficits in aphasic patients. Additionally, also memory functions have been addressed in stroke patients with BCI-based NF interventions [52]. The authors employed a set of relevant neurophysiological indexes that revealed sensitive to training intervention outcomes, therefore useful to be integrated to standard neuropsychological assessment results to evaluate and quantify the changes induced by the BCI-based cognitive rehabilitative intervention.

Burke and colleagues [53] used intracranial EEG (iEEG) in neurosurgical patients to detect theta and alpha oscillations which correlate with optimal memory encoding, thus using them to trigger item presentation in a free recall task. This is the first application of iEEG in a BCI to enhance memory functions.

Besides EEG, NF studies based on functional magnetic resonance (MRI) have been shown to produce behavioral changes in schizophrenia and in substance addiction disorders. Several studies investigated the effect of volitional brain regulation of specific areas, such as amygdala and anterior cingulate, on cognition and behavior. Based on this findings, Rana and colleagues [54] investigated the feasibility of applying real-time feedback MRI NF in aging research. In a sample of healthy elderly, they showed that volitionally control of anterior insula during a facial emotion recognition task is associated with increased cognitive flexibility, supporting the efficacy of this approach for cognitive enhancement and training.

Another interesting field of work concerns the use of virtual reality as a therapeutic intervention for neurorehabilitation and its integration with BCI systems [55]. An interesting application of such model is that of Rohani and Puthusserypady [56], who realized a P300 based VR (virtual classroom) system for training attention abilities in ADHD patients. This study is also the first attempt to develop a BCI system for attention training that is based on P300 potential, according to its direct link to attentional and voluntary cognitive activity. The developed system, tested in healthy participants, revealed to be promising, as supported by usability and motivational aspects.

Overall, the presented studies involve increasing levels of complexity and sophistication with respect to traditional NF models: the integration of multidimensional indexes (i.e., neuropsychological, neurophysiological, and behavioral), the realization of engaging and realistic settings for training of cognitive functions, and the use of innovative BCI systems.

## 4. Conclusions

We presented an overview of studies employing different BCI systems with the aim of realizing cognitive assessment or rehabilitation protocols. Main measures and procedures adopted for the described purposes are summarized as follows. With regard to cognitive assessment, the studies presented mainly employed ad hoc designed cognitive tasks, realized according to the characteristics and restrictions of the BCI paradigm adopted [21, 22, 25, 30]. Cipresso et al. [28, 29] used a widely known cognitive test, that is, Verbal Fluency, even if with relevant modifications in administration and scoring methods with respect to the traditional “paper and pencil” version, in order to adapt to the BCI system. Differently, a few authors [23, 24, 27] realized a BCI-based version of a validated and standardized neuropsychological measure of fluid intelligence, that is, the RCPM, with particular attention at maintaining a reasonable adherence to the original test. Both authors highlighted convergent validity of the adapted test with other related paper and pencil measures. Poletti et al. [27] also realized adaptations of other traditional validated neuropsychological tests, by extending the purposes toward the realization of a verbal-motor free comprehensive neuropsychological battery similar to that employed in clinical settings.

According to cognitive rehabilitation, the methods seem more heterogeneous, according both to the different clinical populations involved (i.e., ADHD, healthy elderly, stroke patients, and spinal cord injury) and to the specific target of cognitive interventions (attention, memory, language, and visuospatial abilities). Typically, cognitive rehabilitation relies on a set of tasks and procedures that are more flexible and adaptable than those used for cognitive evaluation purposes, since it is tailored on patients' specific needs and residual capacities. Moreover, the realization of engaging settings for cognitive training, sometimes involving gaming systems, entails the realization of more realistic and interactive protocols that needs to be consistently adopted across several studies for standardization.

With regard to clinical populations recruited, despite BCI application for cognitive assessment mainly addressed clinical populations with physical disabilities up to locked-in conditions (i.e., ALS and MCS), for whom BCI and other assistive technologies were firstly developed, cognitive rehabilitation has been mainly used with target patients of NFT (ADHD, ASD, and cognitive enhancement in the elderly). An emerging field of study concerns BCI application for rehabilitation of language deficit in stroke patients, even if few and heterogeneous findings have been collected as yet.

An interesting finding arising from the present review concerns the limited number of studies addressing the use of BCI for cognitive assessment of patients with physical limitations, despite the clinical and ethical relevance of longitudinal neuropsychological evaluation in neurological disorder, especially in neurodegenerative conditions. Perhaps, the need for an expensive equipment and specific competencies in order to use the system and analyze data is one of the main obstacles in the use of BCI in clinical settings. At present, a widely used BCI paradigm is the visual P300 BCI: even if it requires patients to perform ocular movements and fixation to some extent, several studies demonstrated that it can be employed also with ALS patients in the late stage of the disease where oculomotor abilities are often altered [57, 58]. However, several studies have proposed gaze-independent P300 or SSVEP-based BCIs for patients with DOC who often lose the ability to fixate their gaze in order to overwhelm such clinical and methodological issue [30].

With regard to the use of BCI alongside more traditional NF applications, feedback visualizations in NFT (and biofeedback) paradigms range from controlling a simple bar graph to more complex and realistic visual stimuli. In typical application of NFT, the feedback is not related to the specific meaning of the signals being trained or the expected behavioral outcomes. However, a specific feedback for certain signals being trained might be more effective in promoting behavioral changes by activating specific brain areas. Moreover, the level of motivation involved in performing the task is increased by the sense of agency that the user perceive, that is, its capacity of making something relevant happen. For these purposes, the introduction of new ways of providing feedback and reward within BCI-based NFT, such as virtual reality environments, appears promising to improve efficacy and transferability of learnings to real-life contexts.

Another issue concerning the use of BCI for both cognitive assessment and, more specifically, rehabilitation purposes is that of slow learners, particularly applicable to older adults [54]. The possibility of integrating expensive modalities targeting deep brain region, such as fMRI-based NF, with less cost-intensive NF training methods, that is, EEG, could help in providing a longer training period to such population. An emerging technology, useful for these purposes, is represented by real-time fMRI (rtfMRI) [59]. Even if this approach has been poorly investigated with randomized clinical studies, its potential application in combination with other technologies deserves further consideration.

To conclude, some emerging challenges arise from the present review and represent possible relevant targets of future investigation within the field of BCI use for clinical purposes. In particular:

1. the possibility of bringing BCI-based training into patient's home, by developing low-cost and portable systems, in order to provide more intensive, effective, and long-term treatments of cognitive functions;
2. in relation to the previous point, the improvement of usability (simplification of procedures) and customizability of BCIs to users' characteristics and cognitive capacities; in particular, the issue of usability investigation has not been detailed in the present review, but represents a relevant aspect when dealing with clinical populations and their families [7];
3. the investigation about outcomes of BCI-based cognitive interventions, with respect to brain functional changes and reorganization; in particular, the use of quantitative measures, such as fMRI and EEG, to be integrated with behavioral and neuropsychological findings, will help to better clarify the efficacy and impact of training protocols, as also suggested by recent reviews [60, 61];
4. the improvement in realization of BCI-based neuropsychological tests, to be validated in clinical populations against gold standard measures; in particular, such approach should take into consideration the simplification of procedures for tests' administration and a limited number of items composing each test, in order to reduce cognitive effort and interference in the detection of patients' cognitive profiles; according to these increased usability and reliability components, several aspects of cognition should be involved in BCI-based assessment protocols, in order to meet clinical and ethical needs involved in neurodegenerative disorders.

Overall, even with some limitations due to technical and methodological issues, literature on BCI highlights promising findings in both cognitive assessment and training contexts, thus promoting innovative BCI-based applications for neurorehabilitation settings and aging research.

## Figures and Tables

**Table 1:** BCI applications to cognitive assessment.

Study (year)	Signals/EEG-based paradigms	Sample	Measure (test)	Tested Variables	Patients' training required (yes/no)
Iversen & coll. (2008a)	SCPs	2 late-stage ALS patients	Delayed matching-to-sample task	Accuracy (% of correct responses)	Yes
Iversen & coll. (2008b)	SCPs	1 late-stage ALS patient	Conditional-associative learning task	Accuracy (% of correct responses)	Yes
Perego & coll. (2011)	SSVEP	19 healthy subjects	RCPM, CFIT	Total scores and total execution time for tests; total moves and bit-rate for BCI performance	Yes
Perego & coll. (2011)	SSVEP	26 patients with different neurological diseases (cerebral palsy, dystrophies and paresis)	RCPM	Total scores and total execution time for tests; bit-rate for BCI performance	Yes
Cipresso & coll. (2012)	P300 ERP	8 healthy subjects	Phonemic and Semantic VF test (modified version); psychological and usability questionnaire	BCI classification accuracy; execution time and errors at VF test; questionnaires' scores	No
Cipresso & coll. (2013)	P300 ERP	8 healthy subjects and one ALS patient	Phonemic and Semantic Verbal Fluency test (modified version), psychological and usability questionnaire	BCI classification accuracy; execution time and errors at VF test; questionnaires' scores	No
Li & coll. (2015)	SSVEP + P300 ERP	11 brain-injured patients (6 VS, 3 MCS, 2 EMCS)	Number recognition, number comparison, mental calculation	Accuracy rate and number of trials for each test	No
Westergren & coll. (2016)	SSVEP	11 healthy subjects	Four cognitive tests resembling WAIS test battery	BCI classification accuracy, ITR and tests' score	No
Poletti & coll. (2016)	P300 ERP	15 moderate-stage ALS patients and 15 healthy controls	Token Test, RCPM, d2 test, MCST	Tests' total scores and execution times	No

SCPs: slow cortical potentials; SSVEP: steady state visually evoked potentials; ERP: event-related potentials; ALS: amyotrophic lateral sclerosis; VS: vegetative state; MCS: minimally conscious state; EMCS: emerged from MCS; RCPM: Raven's colored progressive matrices test; CFIT: Culture Fair Intelligence Test; VF: Verbal Fluency test; WAIS: Wechsler adult intelligence scale; MCST: modified card sorting test; ITR: information transfer rate.

**Table 2:** BCI applications to cognitive rehabilitation.

Study (year)	Signals/paradigms	Sample	Method	Outcome measures
Lim & coll. (2010)	Frontal (Fp1 and Fp2) and parietal (Pz) EEG signals, covering theta, alpha, beta 1, and beta 2 EEG waves	20 ADHD children	Mathematics and English comprehension questions, with the BCI system monitoring attention level	ADHD Rating Scale-IV
Lim & coll. (2012)	Frontal EEG signals (Fp1 and Fp2)	20 ADHD children	Colour Stroop Task during Calibration. Training game (Cogoland). Mathematics and English worksheet	ADHD Rating Scale-IV
Lee & coll. (2013)	Frontal EEG signals (Fp1 and Fp2)	31 healthy elderly	Colour Stroop Task during Calibration. BCI system based on a card-pairing memory game	RBANS. Usability and acceptability questionnaire
Toppi & coll. (2014)	SMRs	2 stroke patients	NF training based on 10 sessions on SMRs	EEG data while performing the Sternberg memory task. Behavioral performance at the Sternberg task. Scores at RAVLT and CBTT
Gomez-Pilar & coll. (2014)	SMR-EEG	40 healthy elderly	NF training consists in imagery motor exercises combined with memory and logical relation tasks	Luria–AND test
Burke & coll. (2015)	iEEG theta and alpha oscillations	14 neurosurgical patients with medication-resistant epilepsy	Individual prestimulus electrode fluctuations used to modulate memory performance	BCI and standard free recall episodic memory task
Lee & coll. (2015)	Frontal EEG signals (Fp1 and Fp2)	39 healthy Chinese-speaking elderly	Colour Stroop Task during Calibration. BCI system based on a card-pairing memory game	Repeatable Battery for the Assessment of Neuropsychological Status (RBANS). Usability and acceptability questionnaire
Rohani & Puthusserypady (2015)	P300 ERP	6 healthy young participants (24–32 years)	Two oddball attention tasks, targeting visual attention and discrimination, performed within a 3D Virtual Classroom	Average error rate in detecting P300 by the classifier
Salisbury & coll. (2015)	EEG (not further specified)	A 25-year-old man with spinal cord injury	Training session with cube rotation and manipulation paradigm presented on a laptop computer, followed by BCI trial (Emotive EEG gaming system)	Screening measures related to cognition, psychological disposition and pain
Salisbury & coll. (2016)	EEG (not further specified)	25 participants (18–64 years) with traumatic or nontraumatic spinal cord injury	Training session with cube rotation and manipulation paradigm presented on a laptop computer, followed by BCI trial (Emotive EEG gaming system)	Screening measures related to cognition, psychological disposition and pain
Gomez-Pilar & coll. (2016)	SMR-EEG	63 healthy elderly	NF training designed for training motor imagery that implies ERS/ERD of alpha and beta frequency bands in the EEG	Luria-AND test
Kim & Lee (2016)	SMR and mid-beta waves of Fp1 and Fp2	20 children with cerebral palsy	BCI-FES group versus FES control group	Sensorimotor rhythms (SMR) and middle beta waves (M-beta)
Kleih & coll. (2016)	P300 ERP	5 stroke patients with aphasia	Visual P300 speller paradigm. TAP to predict spelling success	BCI usability (visual analog scale) and spelling performance (accuracy)
Rana & coll. (2016)	fMRI bold response	8 healthy adults (age > 61 years old)	rtfMRI approach to train participants to upregulate anterior insula during a facial emotion recognition task	Average percentage change in the BOLD signal and DCCS scores
Musso & coll. (2017)	Auditory ERP	20 healthy subjects and 1 aphasic stroke patient	Word ERP responses to 6 bisyllabic words recorded with an auditory BCI	Average target and nontarget ERP responses

EEG: electroencephalogram; SMRs: sensory motor rhythm; iEEG: intracranial EEG; ERP: event-related potentials; SMR: sensorimotor rhythms; FES: functional electrical stimulation; fMRI: functional magnetic resonance imaging; ADHD: attention-deficit/hyperactivity disorder; ASD: autism spectrum disorder; RBANS: repeatable battery for the assessment of neuropsychological status; TAP: attention performance test; Luria–AND test: Luria adult neuropsychological diagnosis (AND) test; DDCS: dimensional change card sort; rtfMRI: real-time fMRI; RAVLT: Rey auditory verbal learning test; CBTT: Corsi block tapping test; NF: neurofeedback.
